# Developing Y‐Branched Polymer Acceptor with 3D Architecture to Reconcile Between Crystallinity and Miscibility Yielding >15% Efficient All‐Polymer Solar Cells

**DOI:** 10.1002/advs.202200864

**Published:** 2022-05-20

**Authors:** Jingjing Ji, Lei Zhu, Xia Xiong, Feng Liu, Ziqi Liang

**Affiliations:** ^1^ Department of Materials Science Fudan University Shanghai 200433 China; ^2^ School of Chemistry and Chemical Engineering Frontiers Science Center for Transformative Molecules In Situ Center for Physical Science and Center of Hydrogen Science Shanghai Jiao Tong University Shanghai 200240 China; ^3^ School of Chemical and Environmental Engineering Shanghai Institute of Technology Shanghai 201418 China

**Keywords:** all‐polymer solar cells, crystallinity, miscibility, polymer acceptor, Y‐branched

## Abstract

In all‐polymer solar cells (all‐PSCs), there remains such a dilemma that obtains good miscibility and crystallinity simultaneously. Herein a new family of Y‐shape polymer acceptor, namely PYTT is developed, which is copolymerized from Y6 and benzotrithiophene units in three‐way directions. Benefiting from its high‐density end‐chains and extended *π*‐conjugation thanks to highly‐branched 3D architecture, PYTT displays better organic solubility despite much higher molecular weights, larger crystallinity, and tighter *π*‐stacking than the linear counterpart—PYT comprising Y6 and thiophene moieties, while showing identical optical absorption yet threefold higher photoluminescence intensity. In PYTT blend film with PM6 polymer donor, the interpenetrating nano‐fibrillar structures are formed with well‐intermixed polymeric domain sizes close to the exciton diffusion length, which is greatly conducive to exciton dissociation and charge transport in device. Consequently, PYTT‐based all‐PSCs exhibit all increased photovoltaic parameters, yielding a decent power conversion efficiency of 15.60%, which is ≈20% enhancement over PYT‐based device, along with low nonradiative loss of 0.221 meV.

## Graphical abstract

Y‐branched polymer acceptor—PYTT featuring 3D highly branched structure displays good organic solubility yet strong crystallinity. When paired with PM6 donor, the reduced domain size is close to the exciton diffusion length in blend film. Thus, PYTT‐based all‐polymer solar cells exhibit an impressive power conversion efficiency of 15.60% and its high tolerability on the active layer thickness along with good photostability.

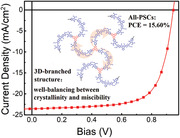

## 1 Introduction

All‐polymer solar cells (all‐PSCs) comprising both polymeric donor and acceptor have attracted ever‐increasing attention thanks to their superior thermal stability, photostability, and mechanical stability when compared to small‐molecule acceptor based organic solar cells (SMA‐OSCs).^[^ ^1^, ^2^, ^3^, ^4^, ^5^ ^]^ In recent years, extensive researches in all‐PSCs have resulted in the best power conversion efficiencies (PCEs) beyond 14%.^[^ ^6^, ^7^ ^]^ The rapid developments of all‐PSCs have great benefits from polymerized small‐molecule acceptors (PSMAs),^[^ ^8^ ^]^ optimized blend film morphology via a combination of solvent additive and thermal annealing,^[^ ^6^ ^]^ and pseudo‐bi‐layered device configuration.^[^ ^9^ ^]^ Remarkably, the last 2 years have witnessed the outstanding advances of the emerging 2,2′‐((2Z,2′Z)‐((4,4,9,9‐tetrahexyl‐4,9‐dihydro‐s‐indaceno[1,2‐b:5,6‐b′]dithiophene‐2,7‐diyl)bis(methanylylidene))bis(3‐oxo‐2,3‐dihydro‐1H‐indene‐2,1‐diy lidene))dimalononitrile (IDIC) and (2,2′‐((2Z,2′Z)‐((12,13‐bis(2‐ethylhexyl)‐3,9‐diundecyl‐12,13‐dihydro‐[1,2,5]thiadiazolo[3,4‐e]thieno[2,″3″:4′,5′]thieno[2′,3′:4,5] pyrrolo[3,2‐g]thieno [2′,3′:4,5]thieno[3,2‐b]indole‐2,10‐diyl)bis(methanylylidene)) bis(5,6‐difluoro‐3‐oxo‐2,3‐dihydro‐1H‐indene‐2,1‐diylidene)) dimalononitrile) (Y6)‐series based PSMAs that feature low optical bandgaps (*E*_g_^opt^s) of 1.4−1.5 eV and intense absorption in the range of 600–900 nm. For instance, Huang and colleagues synthesized such a PSMA of benzothiadiazole (BT)‐based PJ1 polymer by co‐polymerizing Y6 derivative bearing benzothiadiazole (BT) moiety along with a single thiophene *π*‐unit. When matched with PBDB‐T donor, the PJ1−based all‐PSC device exhibited a high extinction coefficient of 1.39 × 10^5^ cm^−1^, yielding an excellent PCE of 14.4% with a prominent short‐circuit current density (*J*_SC_) of 22.3 mA cm^−2^.^[^ ^7^ ^]^ By substituting BT core in PJ1 with benzotriazole (BTz) moiety, Fu et al. presented another kind of PSMA, that is, PZT‐*γ*. After sufficient purification, the resulting high regiospecificity avoided the formation of isomers during polymerization, which endowed PZT‐*γ* with broader and stronger light absorption, longer chain extension, and higher electron mobility than other isomeric polymers, leading to a noticeably high *J*_SC_ of 24.7 mA cm^−2^ along with a small energy loss of 0.51 eV and hence a record‐high PCE of 15.8%.^[^ ^10^ ^]^ Nevertheless, such all‐PSCs were found to be very sensitive to molecular weight (M.W.) of PSMA. Therefore, Min and coworkers developed a series of PYT polymers with shorter *N*‐alkyl chains in derivative Y6‐unit relative to those of PJ1 to investigate the M.W. effect on the photovoltaic performance. It was unraveled the medium number‐averaged M.W. denoted as *M*_n_ (=12.3 kDa) PYTs attained a trade‐off between ordered domain arrangement and suitable phase‐separation in the blend film with PM6 donor, which led to a high fill factor (FF) of 64%.^[^ ^11^ ^]^ Despite a multitude of efforts on Y‐series PSMAs, their all‐PSCs still lag behind SMA‐OSCs whose reported efficiencies have now approached 18%.^[^ ^12^, ^13^ ^]^ This essentially lies in a big hurdle long‐existed among all‐PSCs such that an entropy contribution to the Gibbs free energy (Δ*G*) is significantly reduced in all‐polymer blends, which largely suppresses the miscibility of two different polymers.^[^ ^14^, ^15^ ^]^ In principle, the higher M.W. of polymer is, the more face‐on orientations concurrent with better crystallinity are formed,^[^ ^16^, ^17^ ^]^ which is beneficial for charge transport in all‐PSC devices. However, the high M.W. reduces solubility in organic solvents and causes large‐scale phase‐segregation between polymer donor and acceptor domains,^[^ ^11^ ^]^ which hinders exciton dissociation at the donor/acceptor (D/A) interfaces and results in severe geminate recombination.^[^ ^18^ ^]^

Herein we propose and explore such a kind of spatially structured acceptor polymers—branched *π*‐conjugated polymers (BCPs) that intend to effectively tackle the above‐mentioned morphological issues. BCPs featuring 3D architectures differ from their conventional linear counterparts,^[^ ^19^ ^]^ in that BCPs are intrinsically stretched in all directions and hence tend to form globular structures and provide large internal space and high‐density end‐chains,^[^ ^20^, ^21^ ^]^ both of which guarantee the sufficient contacts with donor polymers, in particular those linear ones. Moreover, BCPs can effectively mitigate the entanglements and aggregation among polymer chains, ensuring good solubility in organic solvents and holding good promise on high‐M.W. BCPs, which generate highly crystalline, pinhole‐free, and thick films.^[^ ^22^, ^23^ ^]^ Consequently, when applied to the BHJs of all‐PSCs, such BCPs can effectively promote the morphological compatibility of polymer blends and aid in the formation of interpenetrating networks, which is greatly conducive to exciton dissociation and charge transport.

In this contribution, we designed and synthesized such a Y‐branched polymer acceptor—PYTT with three extended arms via Stille coupling based polycondensation for all‐PSCs. Compared to its linear as‐synthesized counterpart—PYT, PYTT exhibits better organic solubility, enhanced crystallinity, high photoluminescence (PL) intensity, and low exciton binding energy (*E*_B_). When blended with benchmark PM6 donor, an incredibly near‐unity quenching PL efficiency is obtained while both significantly improved compatibility and tighter *π*–*π* stacking are found in PYTT‐based blend film. As a result, the PM6:PYTT blend based all‐PSC device exhibits all simultaneously enhanced photovoltaic parameters, leading to an impressive PCE of 15.60% with a lower nonradiative loss.

## 2 Results and Discussion

**Figure 1:**
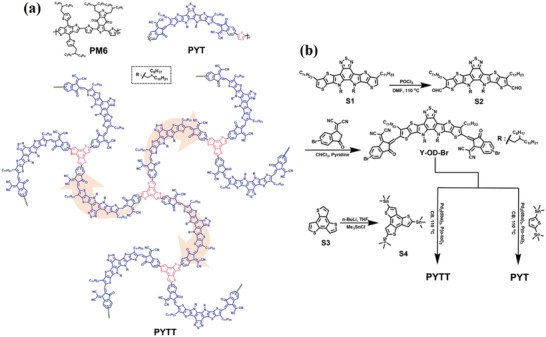
a) Molecular structures of PM6 polymer donor, PYT, and PYTT polymer acceptors and b) synthetic routes of PYT and PYTT.

The molecular structures and synthetic procedures of two polymer acceptors—PYT and PYTT—are depicted in **Figure** **1**. According to high‐temperature gel permeation chromatography (GPC) measurement, PYTT displays a higher *M*_n_ of 28.2 kDa and narrower polydispersity index (PDI) of 1.5 than PYT (*M*_n_ = 13.0 kDa and PDI = 1.8) as shown in Figure S1, Supporting Information and **Table** **1**. Compared to thiophene monomer possessing two reaction sites, the cross‐coupling polymerization products of S2 bearing three identical sites with YOD‐Br may cause structural uncertainty. However, given that all reaction sites of S2 possess the same reaction activity along with the efficient Stille coupling method, there is a very high probability that all three sites of S2 are reacted. Unfortunately, we are unable to determine how exactly the two monomer units are connected in Y‐branched polymer, which can be however rationalized qualitatively by the ensuing high *M*_n_ and narrow PDI, indicating little variation on the PYTT structures. In addition, it is found that PYTT displays a high decomposition temperature (*T*_d_: 5% weight loss) of 340 °C, which is nearly identical to PYT (346 °C) as shown in Figure S2, Supporting Information, suggesting Y‐branched PYTT possesses as high thermal stability as the linear PYT analogue, although Y‐branched or even hyper‐branched polymers are generally regarded to be less stable than the linear analogues. Besides, both polymers can be readily dissolved in common organic solvents such as chloroform (CF) and chlorobenzene (CB) at room temperature, yet high*‐M*_n_ PYTT shows remarkably better solubility than PYT (Figure S3, Supporting Information)—a feature of such branched polymeric 3D architectures with high‐density end‐chains that are distinct from linear analogues.

**Table 1:** Molecular weights, optical, and electrochemical characteristics of PYT and PYTT

*λ*_max_ [nm]						
Polymer	*M*_n_ [kDa]	PDI	Film	$E_{g}^{\mathrm{opt}}$ ^a)^ [eV]	HOMO^b)^ [eV]	LUMO^b)^ [eV]
PYT	13.0	1.80	800	1.44	−5.60	−3.77
PYTT	28.2	1.54	800	1.44	−5.67	−3.80

^a)^ Calculated from the absorption onset of the films;

^b)^ Estimated from the oxidation/reduction onset of the CV curves.

**Figure 2:**
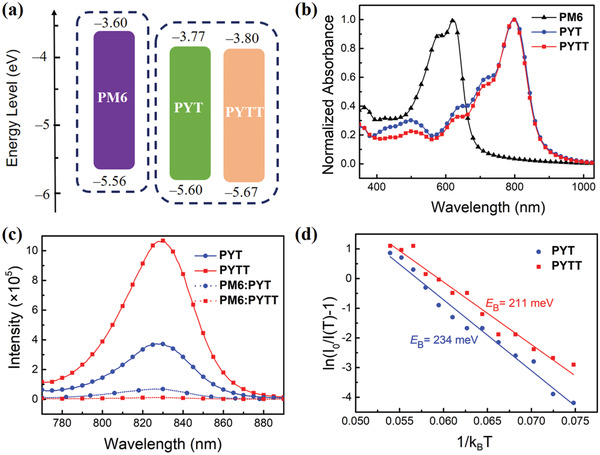
a) Energy level diagram and b) optical absorption spectra of PM6, PYT, and PYTT in thin films, c) PL spectra of PYT, PYTT films, as well as the PM6:PYT and PM6:PYTT blend films, and d) exciton binding energy fitting spectra of PYT and PYTT.

The highest occupied molecular orbital (HOMO) and the lowest unoccupied molecular orbital (LUMO) energy levels of PYT and PYTT were measured by cyclic voltammetry (CV) as shown in Figure S4a, Supporting Information. As summarized in **Figure** **2**a and Table 1, the HOMO/LUMO energy levels of PYTT are estimated to be −5.67/−3.80 eV, close to PYT (−5.60/−3.77 eV). The HOMO levels of both acceptors are slightly deeper than that of PM6 donor, which aids to reduce energy loss in device. In addition, the normalized UV−vis absorption spectra of PYT and PYTT were acquired in both dilute CF solution and thin‐films as shown in Figure 2b and Figure S4b, Supporting Information, and the parameters extracted from the spectra are listed in Table 1. PYT and PYTT show the same major absorption peak centered at 750 nm in solution, whereas both polymer thin films exhibit the bathochromic absorption with the nearly same maximum absorption band at 800 nm, indicative of the closer *π*‐stacks among the conjugated backbones. The corresponding *E*_g_^opt^s of PYT and PYTT films are identical and determined to be 1.44 eV. Both PSMAs present the optical absorption complementary to PM6 donor, which is desirable for solar photon harvesting.

As shown in Figure 2c, the PYTT thin film exhibits a PL intensity of 1.1 × 10^6^, threefolds higher than analogous PYT (3.7 × 10^5^) in the case of identical film thickness, which is attributed to the remarkably 3D extended *π*‐conjugation of highly branched PYTT. When paired with PM6 donor, the PYTT‐based blend film shows a surprisingly higher PL quenching efficiency of 98.75% than that of PYT (81.62%), suggesting an occurrence of highly efficient hole transfer from PYTT to PM6. The almost completely quenching of PYTT by PM6 in PL spectra presumably originates from the decrease in exciton *E*_B_ induced by noticeably more *π*‐delocalized structures in PYTT relative to PYT. This prompts us to further perform temperature‐dependent PL measurement on PYT and PYTT films to determine *E*_B_ values. As shown in Figure S5a−d, Supporting Information, when the temperature varies from 160 to 215 K, the PL intensities of both polymers decline; in contrast, by rising from 215 to 260 K, both increase monotonically in intensity, which is for the first time observed in analogous *π*‐conjugated D–A copolymers. The *E*_B_ values are obtained by fitting the temperature‐varying PL intensities, *I*(*T*), with the Arrhenius equation of *I*(*T*) = *I*_0_/(1 + *A* exp (−*E*_B_/*k*_B_ *T*)) where *T* is the temperature, *I*_0_ is the PL intensity at the lowest temperature, *A* is a constant, and *k*_B_ is the Boltzmann constant (Figure 2d).^[^ ^24^, ^25^ ^]^ As a result, PYTT presents a significantly lower *E*_B_ (211 meV) than PYT (234 meV), giving rise to more efficient exciton dissociation upon photo‐excitation,^[^ ^26^ ^]^ which is in accord with the room‐temperature PL quenching results.

**Figure 3:**
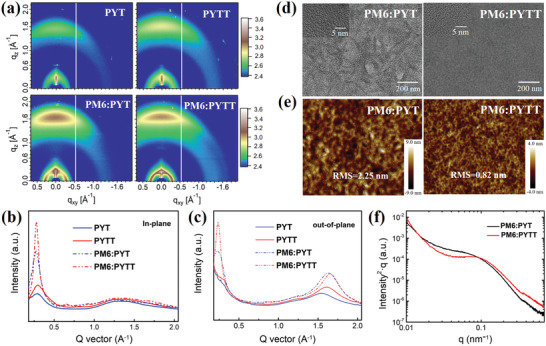
a) GIWAXS profiles of the neat PYT, PYTT films and their blend films, and the corresponding line‐cuts of b) in‐plane and c) out‐of‐plane orientations; d) HR‐TEM images, e) tapping‐AFM height images, and f) RSoXS profiles of the blend films.

The grazing‐incidence wide‐angle X‐ray scattering (GIWAXS) technique was then employed to probe and compare molecular packing and orientation behaviors in thin films of neat and the blend with PM6. As shown in **Figure** **3**a–c, the PYT and PYTT films show (100) lamellar peaks at 0.28 and 0.30 Å^−1^ in the in‐plane (IP) direction, which correspond to the interchain distances (*d*‐spacings) of 22.43 and 20.93 Å, respectively. The (010) peaks of the PYT and PYTT films are located around 1.58 and 1.62 Å^−1^, which reflect *π*–*π* stacks in the out‐of‐plane (OOP) direction with *d*‐spacings of 3.97 and 3.87 Å, respectively. This indicates the neat thin films of PYTT and PYT both adopt preferential face‐on orientations. In addition, the crystallinity coherent length (CCL) of (010) peak in the OOP direction is calculated to be 11.65 Å for PYT and 14.31 Å for PYTT. Besides, the intensities of *π*‐stacks and lamellar peaks of PYTT film are significantly higher than those of PYT film, confirming that PYTT shows higher crystallinity, mostly ascribed to the larger M.W. On the other hand, the blend films of PYT and PYTT with PM6 maintain the predominant face‐on orientations. Moreover, the (010) peak of PYTT‐based blend film is found around 1.64 Å (*d*‐spacing = 3.83 Å) with a CCL of 21.05 Å in the OOP direction, which is markedly increased relative to that of PYT‐based blend film (that is, 1.61 Å^−1^, *d*‐spacing = 3.90 Å and CCL = 18.27 Å), suggestive of a closer molecular packing in PYTT‐based blend film. The intermolecular packing is also correlated intimately with thin film density, which can be determined from X‐ray reflectometry (XRR) profiles (Figure S6, Supporting Information).^[^ ^27^, ^28^ ^]^ XRR characterization is so sensitive to thin film roughness that the larger surface roughness, the lower XRR intensity, and hence the stronger background noise. Therefore, the surface roughness of the film is required to be small enough when using XRR to characterize the film density. As a consequence, the XRR peak of neat PYT film is not prominent due to the larger roughness (Figure S6a, Supporting Information) and thus the film density of PYT cannot be accurately obtained. In contrast, the density of neat PYTT film, PYT‐ and PYTT‐based blend films are determined to be 2.08, 0.95, and 2.10 g cm^−3^, respectively. The PYTT‐based blend film shows a much higher density than that of PYT, further supportive of its noticeably compact packing, which can effectively ameliorate charge transport in the active layer of device.

In order to further distinguish the fine microstructures between PYT‐ and PYTT‐based blend films, high‐resolution transmission electron microscopy (HR‐TEM) and tapping‐mode atomic force microscopy (tappinig‐AFM) were utilized to characterize the bulk morphology and surface topography, respectively. As shown in Figure 3d,e and Figure S7, Supporting Information, the PYT‐based blend film shows over‐sized phase‐segregation coupled with a root‐mean‐square (RMS) surface roughness of 2.25 nm. In contrast, the PYTT‐based blend film displays a smoother surface with a drastically reduced RMS down to 0.82 nm, indicative of superior compatibility between two polymers, which benefits from the linear molecule chains of PM6 interpenetrated within the 3D quasi‐network structures of PYTT. Such a striking difference in surface roughness correlates closely with a significant decrease of XRR intensity of PYT‐ relative to PYTT‐based blend film as shown in Figure S6, Supporting Information. On the other hand, the miscibility of donor and acceptor polymers was evaluated by Flory−Huggins interaction parameters (i.e., *χ*_D−A_) as calculated from an equation of $\chi_{D-A}=K\left(\sqrt{\gamma_{D}}+\sqrt{\gamma_{A}}\right)$ ^2^,^[^ ^29^ ^]^ where *γ*_D_ and *γ*_A_ represent the surface energy of polymer donor and acceptor, respectively, and *K* is the constant. As a result, the PM6:PYTT blend displays a lower *χ*_D−A_ of 0.556 K than the PM6:PYT blend (0.720 K) as shown in Figure S8 and Table S1, Supporting Information(SI), suggesting a better miscibility of the former. Consequently, the well‐distributed interpenetrating nano‐fibrillar structures are observed in PYTT‐based blend film, indicative of better‐connected domains. We further carried out resonant soft X‐ray scattering (RSoXS) experiments to analyze the phase separation of BHJ films. From RSoXS profiles shown in Figure 3f, the domain sizes of 76.45 and 36.14 nm are determined for the PM6:PYT and PM6:PYTT blend films, respectively. The reduced domain size in the PM6:PYTT blend film is close to the exciton diffusion length (i.e., 10−20 nm) in the active layer, which is well correlated with the interpenetrating nano‐fibrillar structures as seen from TEM imaging. In short, both suitable sized domains and high inter‐connectivity in PM6:PYTT blend film favor charge separation and transport as well as reduce nonradiative recombination in device.

**Figure 4:**
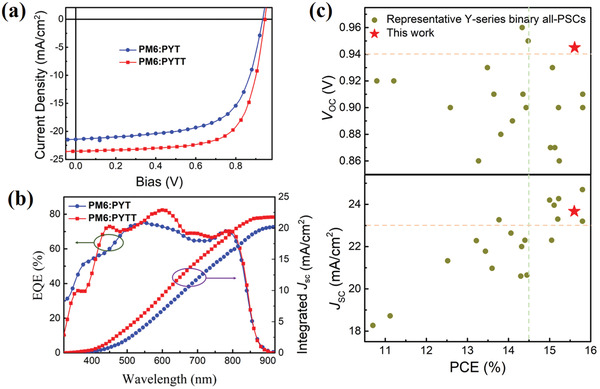
a) *J*–*V* curves and b) EQE and integrated *J*_SC_ spectra. c) A comparison of this work to the reported Y‐series binary all‐PSCs in literature by *V*_OC_ and *J*_SC_ versus PCE.

We fabricated all‐PSC devices based on a conventional architecture of ITO/PEDOT:PSS/active layer/PDINO/Ag to evaluate the photovoltaic effects of both PSMAs. The optimization procedures of the active layer thickness are displayed in Figure S9, Supporting Information, from which the optimal thickness is about 110 nm, and the photovoltaic parameters are shown in Table S2, Supporting Information. We notice that when the thickness increases above 112 nm, the device efficiency barely changes, which can be ascribable to the excellent 3D‐network growth of PYTT, thus facilitating charge transport in device and also indicating the superior thickness tolerance for all‐PSCs.^[^ ^7^ ^]^ In addition, the processing solvent additives of 1,8‐diiodooctane (DIO), dibenzyl ether (DBE), and 1‐chloronaphthalene (CN) were exploited to optimize the morphology of the active layer, whose density–voltage (*J–V*) curves of the devices along with the detailed photovoltaic parameters are shown in Figure S10 and Table S3, Supporting Information, which determines CN to be the favorite for device performance and it is also in good accordance with the relevant literature.^[^ ^11^ ^]^ The CN‐treated optimal devices exhibit the representative photocurrent *J*–*V* curves as shown in **Figure** **4**a and the corresponding parameters are summarized in **Table** **2**. The PYT‐based device shows a PCE as high as 13.02% along with high *V*_OC_ of 0.934 V, *J*_SC_ of 21.31 mA cm^−2^, and FF of 65.40%, which are comparable to state‐of‐the‐art PYT‐based all‐PSCs in literature.^[^ ^11^, ^30^, ^31^, ^32^ ^]^ In contrast, PYTT‐based device yields a slightly increased *V*_OC_ of 0.945 V yet significantly improved *J*_SC_ of 23.67 mA cm^−2^ and FF of 69.80%, thereby achieving a much higher PCE of 15.60%, which is by far among the best reported binary all‐PSCs (see Table S4, Supporting Information). It is worth pointing out that the medium‐*M*_n_ PSMA is the best to acquire good device performance, whereas either too high‐ or too low‐*M*_n_ PSMA deteriorates device performance.^[^ ^31^ ^]^ Also note that the PYTT‐based binary device achieves such a superior efficiency beyond 15% that is positioned among the best comprising the highest‐*M*_n_ Y‐series PSMAs (see Table S4, Supporting Information for detailed comparison).

**Table 2:** Photovoltaic parameters of the optimal PM6:PYT and PM6:PYTT devices

Devices	*V*_OC_ [V]	*J*_SC_ [mA cm^−2^]	*J*_SC_^cal^ [mA cm^−2^]^a)^	FF [%]	PCE [%]
PM6:PYT	0.934	21.31	20.21	65.40	13.02
	(0.934 ± 0.005)	(21.56 ± 0.4)		(65.82 ± 0.85)	(12.88 ± 0.16)^b)^
PM6:PYTT	0.945	23.67	22.13	69.80	15.60
	(0.945 ± 0.004)	(24.10 ± 0.45)		(70.01± 0.59)	(15.40 ± 0.40)^b)^

^a)^ *J*_SC_ values are calculated from the EQE curves;

^b)^ The average parameters were calculated from over eight cells.

The external quantum efficiency (EQE) spectra are presented in Figure 4b and the integrated *J*_SC_ values calculated from the EQE spectra agree well with those obtained from the *J*−*V* curves. The EQE photon response range of PYTT‐based device accords well with their absorption spectra, in particular with a maximum EQE value above 80%. Notably, PYTT‐based device exhibits a higher EQE in the 450−800 nm absorption range than PYT‐based device, indicating significantly better charge photogeneration and separation, which is responsible for significantly enhanced *J*_SC_. In order to better comprehend the merits of PYTT, Figure 4c summarizes and compares with the reported Y‐series binary all‐PSCs with regard to *V*_OC_ and *J*_SC_ versus PCE values, among which the overlapped beige and green regions are representative of the simultaneously enhanced *J*_SC_ and *V*_OC_, in particular highlighting PYTT‐based all‐PSCs with very high *J*_SC_ and surprisingly the highest *V*_OC_. Additionally, we comparatively evaluated the photo‐stabilities of PYTT‐ and PYT‐based all‐PSCs with encapsulation under ambient conditions. As shown in Figure S11, Supporting Information, the PCEs of PM6:PYT and PM6:PYTT devices under 1 sun light illumination maintain 68% and 69% of their initial values after 600 h, respectively. Additional 20 h light exposure begins to cause the PYT device unstable whereas the PYTT device continue to function and can retain 63% initial PCE at 800 h.

**Figure 5:**
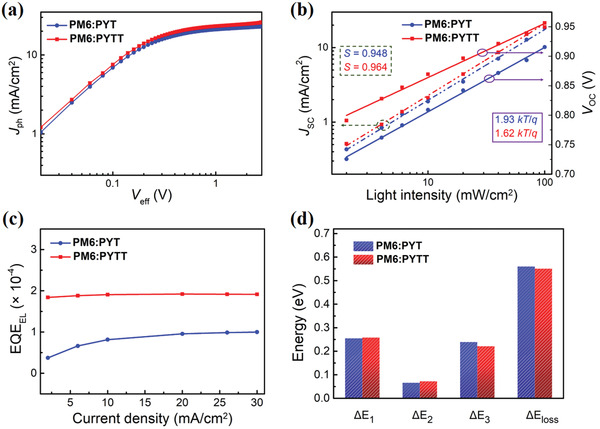
a) *J*_ph_–*V*_eff_ curves, b) light‐intensity dependences of *J*_SC_ (dash line) and *V*_OC_ (solid line), and c) EL quantum efficiencies at a range of injected current densities. d) Analysis of *E*_loss_ into three parts of Δ*E*_1_, Δ*E*_2_, and Δ*E*_3_.

In order to gain a deep understanding the disparity in device performance, the exciton dissociation (*η*_diss_), and the charge collection efficiency (*η*_coll_) are investigated by the plots of the photocurrent (*J*_ph_) as a function of the effective voltage (*V*_eff_)^[^ ^33^ ^]^ shown in **Figure** **5**a. The *η*_diss_/*η*_coll_ are determined to be 97%/81% for PYTT‐based device, which are notably higher than those (93%/71%) of PYT‐based device. The higher the *η*_diss_/*η*_coll_ values are, the more effective exciton dissociation and charge collection are in PYTT‐based device, which accounts for the higher EQE and *J*_SC_ and is also consistent with the above thin film characterization. Besides, to visualize carrier recombination in device, the *J*−*V* curves in the dark are shown in Figure S12, Supporting Information. In comparison to PYT, PYTT‐based device displays lower leakage currents at reverse bias and higher currents as the forward bias is larger than 1.0 V, suggestive of more efficient charge transfer and suppressed carrier recombination in device.^[^ ^34^, ^35^ ^]^ Subsequently, the dependence of *J*_SC_ on light intensity was evaluated to probe the non‐geminate recombination dynamics. The relation between *J*_SC_ and *P*_light_ is expressed by an equation of *J*_SC_ ∝ *P*_light_ *^S^* where *S* indicates the degree of bimolecular recombination.^[^ ^36^ ^]^ As shown in Figure 5b (dash line), the *S* value of PYTT‐based device is found to be 0.964, which is closer to 1 than that of PYT‐based device (*S* = 0.948), indicating that PYTT‐based device effectively retards bimolecular recombination. Furthermore, by plotting the curves of *V*_OC_ versus *P*_light_, the dominant charge recombination mechanism can be further studied.^[^ ^37^ ^]^ Figure 5b (solid line) shows that the slopes are calculated to be 1.93 *kT*/*q* and 1.62 *kT/q* for PYT‐ and PYTT‐based devices, respectively, indicative of less trap‐assisted recombination in PYTT‐based device, which accounts for high *V*_OC_ and reduced energy loss as discussed later. In addition, the charge transport characteristics were assessed by the space‐charge limited current (SCLC) as shown in Figure S13, Supporting Information. The electron and hole mobilities are determined to be *µ*_e_ = 8.3 × 10^−4^ cm^2^ V^−1^s^−1^ and *μ*_h_ = 9.1 × 10^−5^ cm^2^ V^−1^s^−1^ in PYTT‐based device, respectively, leading to a ratio of *µ*_e_/*μ*_h_ = 9.12, which is comparatively more balanced than those of PYT‐based device (i.e., *µ*_e_ = 9.5 × 10^−4^ cm^2^ V^−1^s^−1^, *μ*_h_ = 7.9 × 10^−5^ cm^2^ V^−1^s^−1^ with *µ*_e_/*μ*_h_ = 12.03). This phenomenon combined with the reduced carrier recombination is responsible for both higher FF and *J*_SC_ in device.

To unveil the origins of *E*_loss_ of all‐PSCs, the electroluminescence (EL) spectra were measured under different injected current densities. As shown in Figure S14, Supporting Information, the PYTT‐based device exhibits a notably higher EL intensity of 9.0 × 10^3^ than that of PYT (1.3 × 10^3^), indicative of larger emissive quantum yield for the charge‐transfer states for PYTT‐based device.^[^ ^11^ ^]^ Furthermore, the *E*_loss_ is quantitatively assessed by an equation of *E*_loss_ = *E*_g_ − *qV*_OC_ where *E*_g_ is estimated from the intersection of the EL and EQE curves as shown in Figure S15, Supporting Information. In this respect, the *E*_g_s of PM6:PYT and PM6:PYTT BHJ films are determined to be 1.494 and 1.498 eV, respectively. As a result, the PYTT‐based device displays a relatively lower *E*_loss_ value of 0.553 eV than PYT‐based device (0.560 eV). In order to further interpret how the Y‐branched structures in PYTT aid in the reduction of *E*_loss_, the three parameters that determine *E*_loss_ (also namely, Δ*E*) are analyzed by an equation of $\Delta E=\left(E_{g}-qV_{\mathrm{OC}}^{\mathrm{SQ}}\right)+\left(qV_{\mathrm{OC}}^{\mathrm{SQ}}-qV_{\mathrm{OC}}^{\mathrm{rad}}\right)+\left(qV_{\mathrm{OC}}^{\mathrm{rad}}-qV_{\mathrm{OC}}\right)=\Delta E_{1}+\Delta E_{2}+\Delta E_{3}$.^[^ ^38^ ^]^ Among them, Δ*E*_1_ represents the radiative recombination loss above the optical *E*_g_, and $qV_{\mathrm{OC}}^{\mathrm{SQ}}$ is the maximum voltage from the Shockley−Queisser limit. As shown in **Table** **3**, the PYT and PYTT based devices show the Δ*E*_1_ values of 0.255 and 0.258 eV, respectively, which fall within a range of 0.25−0.3 eV that exists inevitably in solar cells,^[^ ^39^ ^]^ while Δ*E*_2_ is caused by the radiative recombination below the optical *E*_g_, which can be reduced by minimizing the energy level offsets between the donor and acceptor. Therefore, the PYTT‐based device displays a notably higher Δ*E*_2_ of 0.074 eV than PYT (0.066 eV) mainly due to the deeper HOMO energy level. Δ*E*_3_ arises from non‐radiative recombination loss of carriers, which can be decreased by enhancing EQE_EL_to increase *V*_OC_, and is calculated from an equation of Δ*E*_3_ = −*kT* × ln(EQE_EL_) where *k* is the Boltzmann constant and *T* is the absolute temperature. As shown in Figure 5c, the EQE_EL_ value of PYTT‐based device is determined to be 1.93 × 10^−4^, twice higher than PYT‐based device (9.55 × 10^−5^), and hence the Δ*E*_3_ values correspond to 0.221 and 0.239 eV, respectively (Figure 5d). The Δ*E*_3_ value of PYTT‐based device is among the lowest for high‐efficiency binary all‐PSCs in literature. In sum, the above detailed analysis has manifested that the suppressed *E*_loss_ in PYTT‐based device can be mainly attributed to the reduced non‐radiative loss, which is key to raising *V*_OC_, further demonstrating the vitality of conceptual designing such Y‐branched structured polymer acceptor.

**Table 3:** *E*_loss_ analysis of all‐PSCs based on PM6:PYT and PM6:PYTT devices

Acceptors	*E*_g_ [eV]	*qV*_OC_ [eV]	$qV_{\mathrm{OC}}^{\mathrm{rad}}$ [eV]	$qV_{\mathrm{OC}}^{\mathrm{SQ}}$ [eV]	Δ*E* [eV]	Δ*E*_1_ [eV]	Δ*E*_2_ [eV]	Δ*E*_3_ [eV]
PYT	1.494	0.934	1.173	1.239	0.560	0.255	0.066	0.239
PYTT	1.498	0.945	1.166	1.240	0.553	0.258	0.074	0.221

## 3 Conclusions

To conclude, we have for the first time developed such a polymer acceptor, PYTT that features unique Y‐branched 3D structures. Compared to linear counterpart of PYT, PYTT displays a higher degree of crystallinity yet better organic solubility, which is distinct characteristics of highly‐branched polymers. In addition, PYTT shows the identical optical absorption range, yet threefold higher PL intensity, which is surprisingly quenched by a near‐unity in blend film with PM6 donor, indicative of extremely efficient charge transfer, which is responsible for high *J*_SC_ of PYTT‐based device. More importantly, the linear polymer molecules of PM6 are readily interpenetrated into the 3D quasi‐networks of PYTT, ensuring well‐distributed nano‐fibrillar structures in blend film and resulting in efficient exciton dissociation of 97%. The subsequent reduced domain size in the PM6:PYTT blend film yields efficient charge transport and suppressed carrier recombination. In contrast, the PYT‐based blend film suffers from oversized phase‐separation, which leads to inferior exciton dissociation and severe charge recombination. Consequently, the PM6:PYTT blended all‐PSCs exhibit all simultaneously enhanced photovoltaic parameters, delivering an impressive PCE of 15.60%, which is among the best all‐PSCs and highly outperform the PYT‐based device (13.02%). Encouragingly, PYTT‐based all‐PSCs exhibit a remarkably low nonradiative loss of 0.221 meV as well as superior tolerability of PCE on the active layer thickness ranging from 90 to 200 nm and good photostability up to 800 h.

## 4 Experimental Section

### Materials

Chemicals and reagents were purchased from commercial sources and used without further purification unless otherwise indicated. All solvents were dried by the activated molecular sieve and all reactions were performed under a nitrogen atmosphere.

### Synthesis of 2,13‐Bis(2‐octyldodecyl)‐3,9‐Diundecyl‐12,13‐Dihydro‐[1,2,5]thiadiazolo[3,4‐e]thieno[2″,3″:4′,5′]thieno[2′,3′:4,5]pyrrolo[3,2‐g]thieno[2′,3′:4,5] Thieno[3,2‐b]indole‐2,10‐Dicarb‐Aldehyde (S2)

POCl_3_ (0.84 mL, 9.5 mmol) was added dropwise into DMF (2 mL) solution under N_2_ atmosphere at 0 °C. After stirring for 1 h at room temperature, 30 mL CF solution of S1 (1.2 g, 0.94 mmol) was added. The mixture was stirred at ice bath for 1 h and then heated to 70 °C for overnight. After cooling down to room temperature, the system was quenched with water, extracted with CH_2_Cl_2_ for three times, and dried over anhydrous MgSO_4_. After filtration, solvent was removed by rotary evaporation, and the crude product was purified on silica gel chromatography using petroleum ether/CH_2_Cl_2_ (8:1 vol%) to give compound S2 as red solids (1.0 g, 80%).^[^ ^40^ ^] 1^H NMR (400 MHz, CDCl_3_): *δ* 10.13 (s, 2H), 4.62 (d, *J* = 7.7 Hz, 4H), 3.20 (t, J = 7.7 Hz, 4H), 2.03 (s, 2H), 1.98−1.85 (m, 4H), 1.51−0.64 (m,114H).

### Synthesis of Y‐OD‐Br

S2 (1.28 g, 0.94 mmol), IC‐Br (1.08 g, 3.75 mmol), and pyridine (1.5 mL) were dissolved in CF (50 mL). The mixture was stirred at 70 °C overnight under N_2_ atmosphere. After cooling to room temperature, the mixture was poured into methanol. The residue was purified with column chromatography on silica gel using petroleum ether/CH_2_Cl_2_ (1:1 vol%) as the eluent to give a dark blue solid Y‐OD‐Br (1.14 g, 65%).^[^ ^41^ ^] 1^H NMR (400 MHz, CDCl_3_): *δ* 9.18 (d, *J* = 1.3 Hz, 2H), 8.84 (d, *J* = 1.2 Hz, 1H), 8.56 (d, *J* = 8.4 Hz, 1H), 8.03 (s, 1H), 7.85−7.90 (m, 2H), 7.79 (d, *J* = 8.0 Hz, 1H), 4.76−4.87 (m, 4H), 3.22 (t, *J* = 7.7 Hz, 4H), 2.17 (s, 2H), 1.83−1.91 (m, 4H), 1.50−0.74 (m,114H).

### Synthesis of 2,5,8‐Tris(trimethylstannyl)benzo[1,2‐b:3,4‐b′:5,6‐b″]trithiophene (S4)

To a solution of benzo[1,2‐b:3,4‐b′:5,6‐b″]trithiophene 1 (420 mg, 1.70 mmol) in THF (45 mL) at 0 °C was added n‐BuLi (2.5 M solution in hexane, 4.1 mL) dropwise, and the mixture was stirred for 2 h at room temperature. The reaction was subsequently cooled down to −78 °C and then Me_3_SnCl (1.09 g, 6.81 mmol) was added. The mixture was allowed to stir at 0 °C for 4 h. The reaction was quenched with the water (20 mL) and extracted with CHCl_3_ (3 × 30 mL). The mixture was washed with the solution of NH_4_Cl and water, dried over anhydrous MgSO_4_, and concentrated in vacuo. The crude product was re‐suspended with MeOH, sonicated for 10 min, and cooled down at 0 °C. The resulting solid was filtered and washed with cold MeOH to afford white solid product.^[^ ^41^, ^42^ ^] 1^H NMR (400 MHz, CDCl_3_) *δ*: 7.68 (s, 3H), 0.478 (s, 27H); Elemental analysis calculated (%) for C_21_H_30_S_3_Sn_3_: C, 34.33; H, 4.12. Found: C, 34.51; H, 4.01.

### Synthesis of PYT

A mixture of Y‐OD‐Br (93.73 mg, 0.05 mmol), 2,5‐bis(trimethylstannyl)thiophene (20.38 mg, 0.05 mmol), Pd_2_(dba)_3_ (0.92 mg, 0.001 mmol), and P(o‐Tolyl)_3_ (1.22 mg, 0.004 mmol) were added into a Schlenk tube. A 6 mL of anhydrous chlorobenzene was then added to the mixture. The whole system was stirred at 100 °C for 24 h under N_2_ atmosphere. After polymerization, the mixture was cooled down to room temperature and then methanol was added to precipitate and obtain polymer solids. The polymer was purified sequentially by using Soxhlet extractions with a sequence of methanol, petroleum ether, acetone, dichloromethane, and CF for 24 h each, and dried to obtain dark solid polymer (79 mg, yield: 85%).^[^ ^43^ ^]^ *M*_n_ = 13.0 kDa; PDI = 1.80. Elemental analysis calculated (%) for PYT: C, 73.53; H, 7.74; N, 6.24; O, 1.78; S, 10.71. found: C, 73.27; H, 7.87; N, 5.97; O, 2.61; S, 10.28.

### Synthesis of PYTT

Y‐OD‐Br (112.47 mg, 0.06 mmol), 2,5,8‐tris(trimethylstannyl)benzo[1,2‐b:3,4‐b′:5,6‐b″]trithiophene (14.70 mg, 0.02 mmol), Pd_2_(dba)_3_ (3.66 mg, 0.004 mmol), and P(o‐Tolyl)_3_ (4.87 mg, 0.016 mmol) were added into a Schlenk tube. The next steps were the same as those followed for the polymerization of PYT. The dark solid polymer was obtained with 100 mg (the mixed fragments from dichloromethane and CF, yield: 93%). For a further purification, the last CF fractions were collected with a yield of 60%, which were used in this work unless otherwise specified. *M*_n_ = 28.2 kDa; PDI = 1.54.

### Polymer Characterization

High temperature GPC was carried out on a ShimadzuSIL‐20A liquid chromatography instrument using 1,2,4‐trichlorobenzene (TCB) as eluent at 150 °C with polystyrenes as standards. Optical absorption spectra of samples were acquired on Agilent 8453 UV–vis spectrophotometer. Steady‐state PL was measured using a FluoroMax@‐4 spectrofluorometer (HORIBA JOBIN YVON, Inc., Edison, NJ) with the excitation beam at 500 nm. CV measurements were record with a three‐electrode cell under a nitrogen atmosphere in a deoxygenated anhydrous acetonitrile solution of tetra‐*n*‐butylammonium hexafluorophosphate (0.1 m). A platinum disk electrode, platinum wire, and Ag/AgCl electrode were used as a working electrode, a counter electrode, and a reference electrode separately, and the films of polymers were coated on the surface of platinum disk electrode. The CV curves were calibrated using ferrocene/ferrocenium (Fc/Fc^+^) redox couple as an external standard, which was performed at the same conditions as the samples.

### Thin Film Characterization

HR‐TEM imaging was performed on Tecnai G2 F20 S‐Twin microscope at an accelerating voltage of 200 kV. Bruker Dimension Edge atomic force microscope (AFM) in the tapping mode was utilized to image blend film topographies. The height images were gained at a scan rate of 1 Hz using a silicon etched tip, which had a resonance frequency of ≈300 kHz and a spring constant of ≈40 N m^−1^.

### Device Fabrication and Measurements

Solar cell devices were fabricated with a conventional device structure of ITO/PEDOT:PSS/active layer/PDINO/Ag. The ITO‐coated glass substrates were sonicated successively with detergent, deionized water, acetone, and isopropanol, and dried with nitrogen flow. Immediately prior to device fabrication, the substrates were cleaned by oxygen plasma for 20 min. A layer of PEDOT:PSS was then spin‐coated onto the ITO and annealing at 150 °C for 20 min. The active layer was stirred at room temperature for 2 h and was obtained by spinning from CF solution with CN as the processing additive at 3000 rpm for 30 s. The constant weight ratio of donor/acceptor was 1:1 with a total concentration of 14 mg mL^−1^. Subsequently, PDINO in CH_3_OH solution was spin‐coated as electron transporting layer. Finally, Ag (100 nm) was evaporated at a vacuum of ≈1.5 × 10^−4 ^Pa to form the top electrode. The *J*–*V* data were acquired from a Keithley 2400 source‐meter unit. The light *J*–*V* curves were measured under light illumination with a Newport‐Oriel (Sol3A Class AAA Solar Simulator, 94043A) AM 1.5 G light source operating at an intensity of 100 mW cm^−2^. The light intensity was calibrated by a certified Oriel reference cell (91 150 V) and verified with an NREL calibrated, filtered silicon diode (Hamamatsu, S1787‐04). EQE spectra were measured on a commercial EQE set‐up (QE‐R, Enli Technology Co., Ltd). A calibrated silicon diode with a known spectral response was used as a reference. Hole and electron mobilities were attained by using the space charge limited current (SCLC) method.^[^ ^44^ ^]^ The structure of ITO/PEDOT:PSS/active layer/MoO_3_/Ag was used for hole‐only devices and the structure of ITO/ZnO/active layer/ PDINO/Ag was used for electron‐only devices, respectively. The SCLC mobilities were calculated by MOTT–Gurney equation of *J* = 9*ε*_0_ *ε*_r_µV^2^/8*L* ^3^, where *J* is the current density, *ε*_0_ is the dielectric constant of empty space, *ε*_r_ is the relative dielectric constant of active layer materials which is taken to be 3 in the calculation, *μ* is the charge mobility, *V* is the internal voltage in the device, and *L* the thickness of the active layers. *V* can be calculated as *V* = *V*_appl_ − *V*_bi_, where *V*_appl_ is the voltage applied to the devices and *V*_bi_ is the built‐in voltage from the relative work function difference between the two electrodes.

## Conflict of Interest

The authors declare no conflict of interest.

## Supporting information

Supporting Information

## Data Availability

The data that support the findings of this study are available from the corresponding author upon reasonable request.
