# Supplementary material for: Developing Y‐Branched Polymer Acceptor with 3D Architecture to Reconcile Between Crystallinity and Miscibility Yielding >15% Efficient All‐Polymer Solar Cells
Source: Adv Sci (Weinh). 2022 May 20;9(21):2200864. doi: 10.1002/advs.202200864 (PMC9313542; doi:10.1002/advs.202200864)
Supplement: Supplementary file 1 — Supporting Information [file ADVS-9-2200864-s001.pdf]

ADVANCED SCIENCE
Open Access

Supporting Information

for *Adv. Sci.*, DOI 10.1002/advs.202200864

Developing Y-Branched Polymer Acceptor with 3D Architecture to Reconcile Between Crystallinity and Miscibility Yielding >15% Efficient All-Polymer Solar Cells

*Jingjing Ji, Lei Zhu, Xia Xiong, Feng Liu and Ziqi Liang**

Supporting Information

Developing Y-Branched Polymer Acceptor with 3D Architectures to Reconcile Between Crystallinity and Miscibility Yielding >15% Efficient All-Polymer Solar Cells

Jingjing Ji,[1] Lei Zhu,[2] Xia Xiong,[3] Feng Liu[2] and Ziqi Liang[1]*

[*][1]Prof. Z. Liang, J. Ji
Department of Materials Science
Fudan University
Shanghai 200433, China
Email: zqliang@fudan.edu.cn

[2]Prof. F. Liu, Dr. L. Zhu
School of Chemistry and Chemical Engineering
Frontiers Science Center for Transformative Molecules
In-situ Center for Physical Science
and Center of Hydrogen Science Shanghai Jiao Tong University
Shanghai 200240, P. R. China
E-mail: fengliu82@sjtu.edu.cn

[3]X. Xiong
School of Chemical and Environmental Engineering
Shanghai Institute of Technology
Shanghai 201418, China

Results

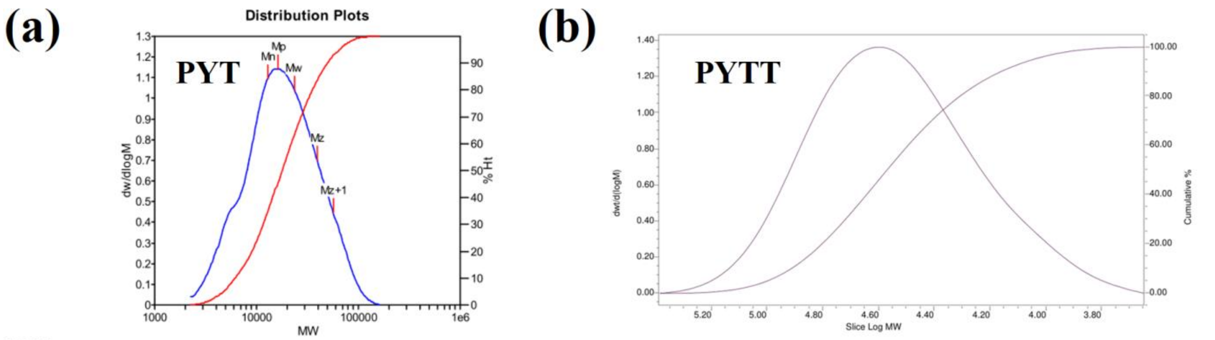

MW Averages

Peak No	Mp	Mn	Mw	Mz	Mz+1	Mv	PD
1	16117	12936	23551	39458	57137	21769	1.82058

	Distribution Name	Mn (Daltons)	Mw (Daltons)	MP (Daltons)	Mz (Daltons)	Mz+1 (Daltons)	Polydispersity	Mz/Mw	Mz+1/Mw
1		28161	43397	39925	61937	81964	1.540995	1.427236	1.888713

Figure S1. The molecular weight distributions of (a) PYT and (b) **PYTT** as determined by GPC.

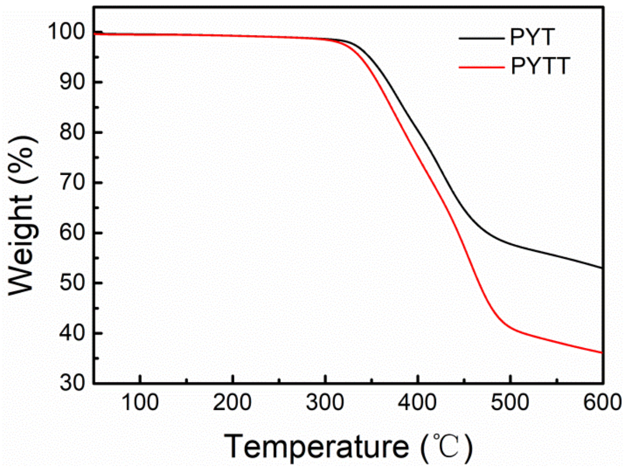

Figure S2. TGA curves of as-synthesized PYT and **PYTT**.

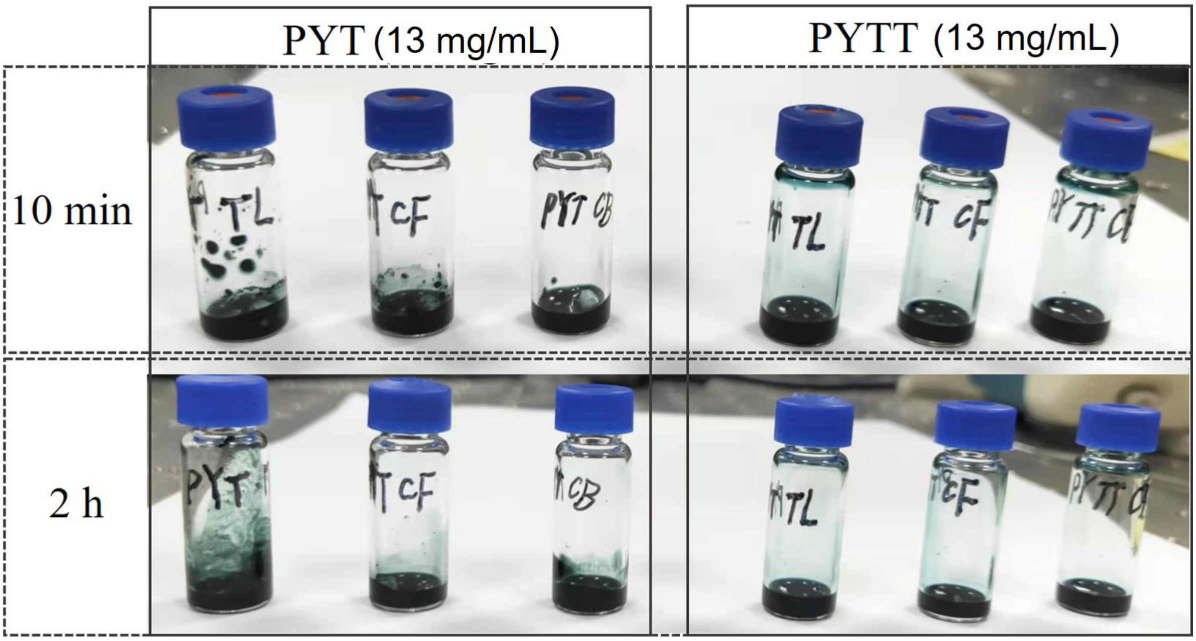

Figure S3. Solubility comparison of both PYT and **PYTT** in different solvents stirred at room temperature. Note TL, CF and CB refer to toluene, chloroform and chlorobenzene, respectively.

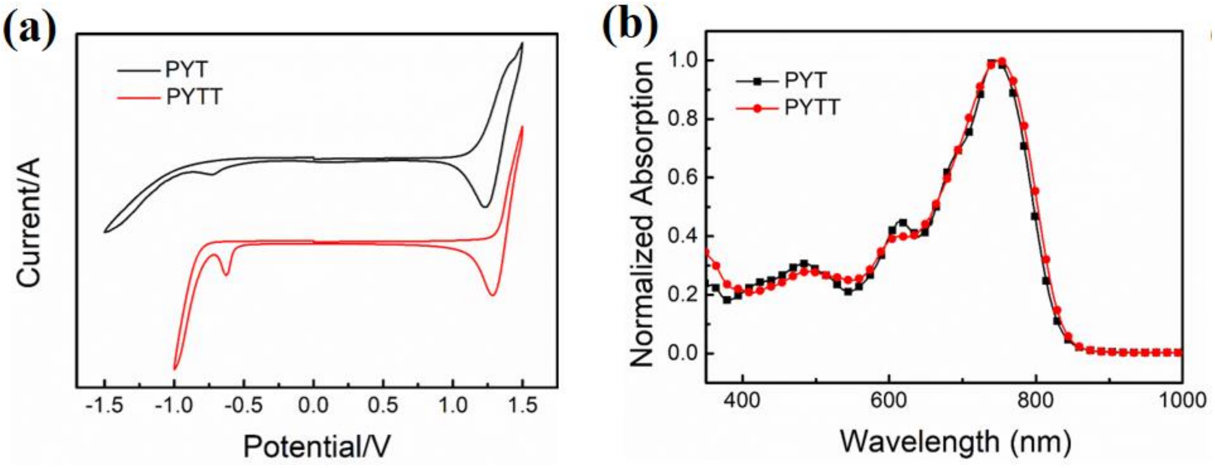

Figure S4. (a) Cyclic voltammetry traces of PYT and **PYTT** in thin films and (b) UV–vis absorption spectra of PYT and **PYTT** in solution.

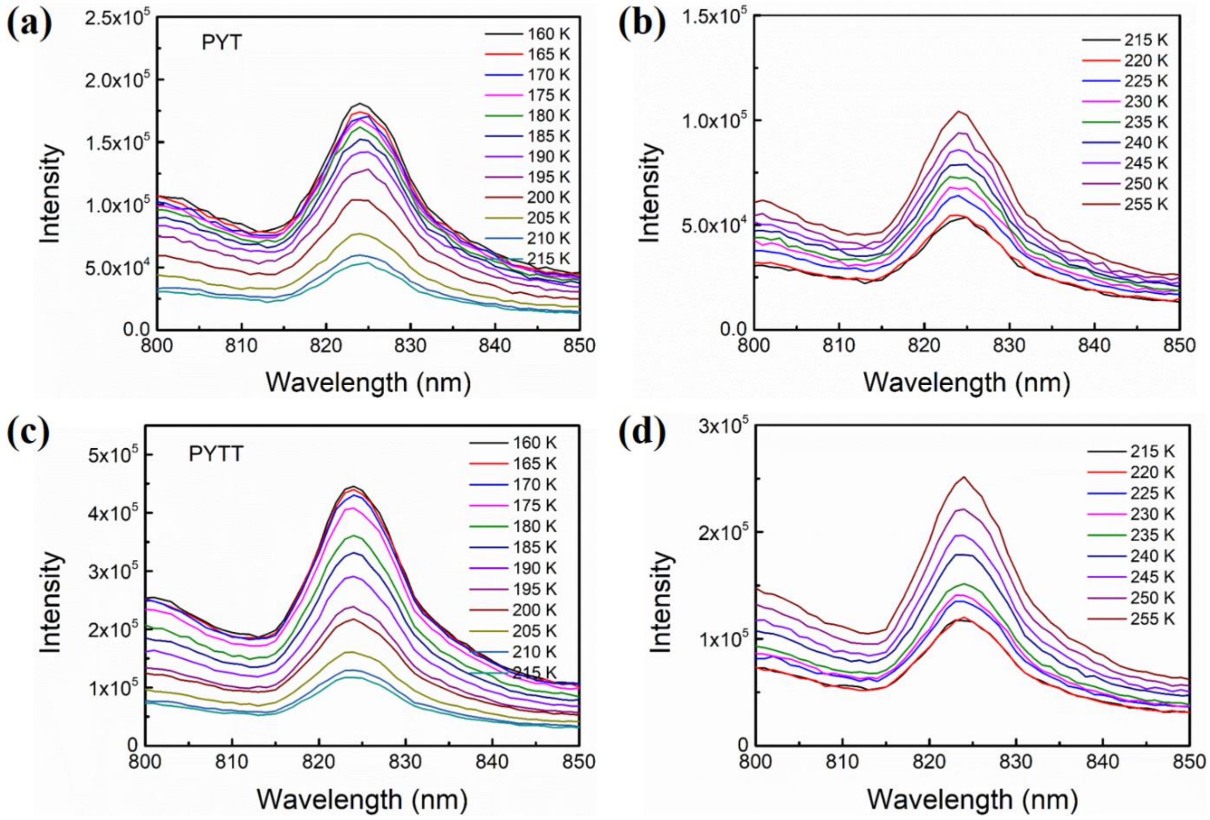

Figure S5. Temperature-dependent PL spectra of (a, b) PYT and (c, d) **PYTT** in thin films.

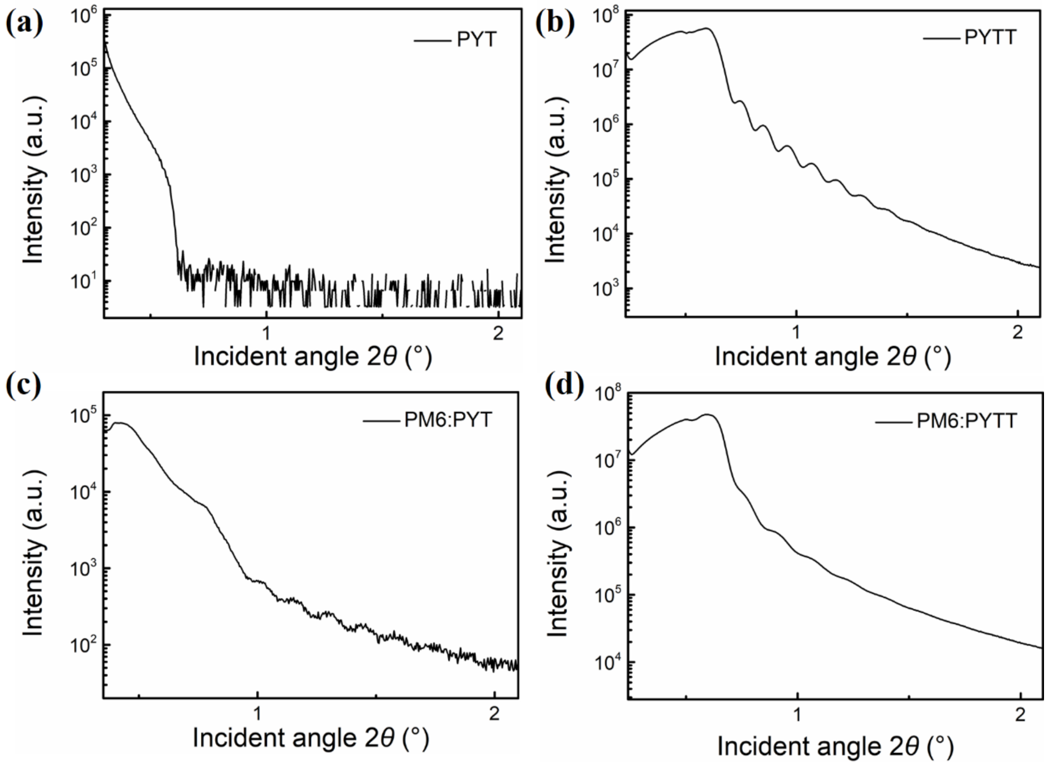

Figure S6. XRR spectra of (a) neat PYT, (b) neat **PYTT**, (c) PM6:PYT and (d) PM6:**PYTT** blend thin films.

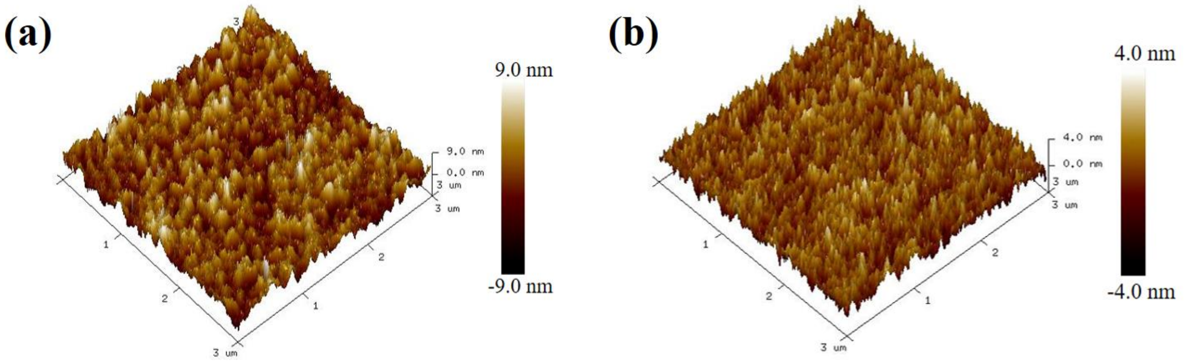

Figure S7. TP-AFM height images of (a) PM6:PYT and (b) PM6:**PYTT** blend films.

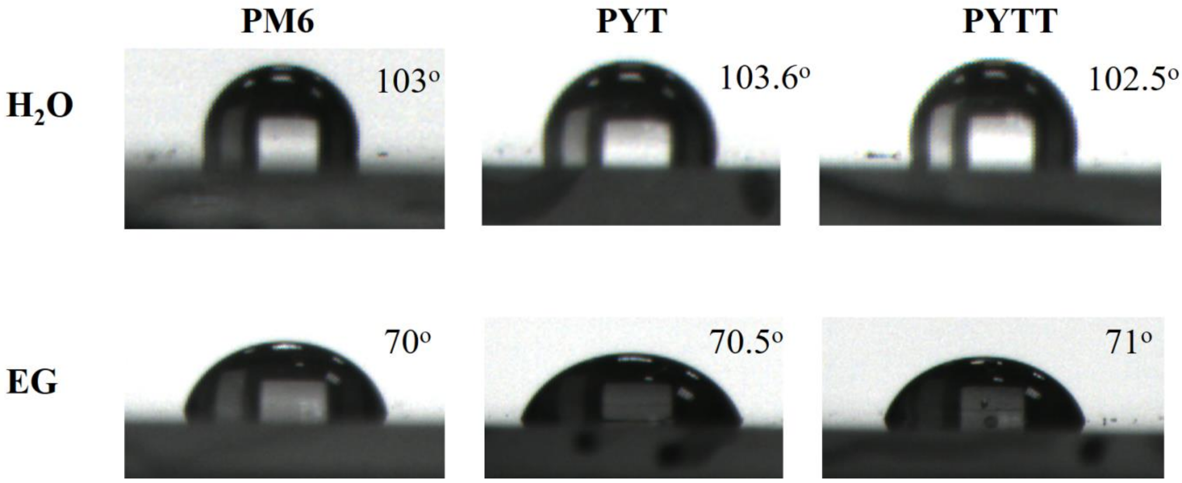

Figure S8. Contact angle measurements of the neat PM6, PYT and **PYTT** films casted from chloroform solution. The measurements were carried out by water (H_2O) and ethylene glycol (EG) as the wetting liquid, respectively.

Table S1. Contact angles and surface free energy parameters of PM6, PYT and **PYTT** films

Film	Contact Angle (°)		γ	$\chi_{donor\text{-}acceptor}$
	H_2O	EG	(mJ m^{-2})	(K)
PM6	103	70	6.005	None
PYT	103.6	70.5	2.566	0.720
PYTT	102.5	71	2.906	0.556

Note: The Flory–Huggins interaction parameter ($\chi_{donor\text{-}acceptor}$) are calculated by $\chi = K(\sqrt{\gamma_{donor}} - \sqrt{\gamma_{acceptor}})^2$.

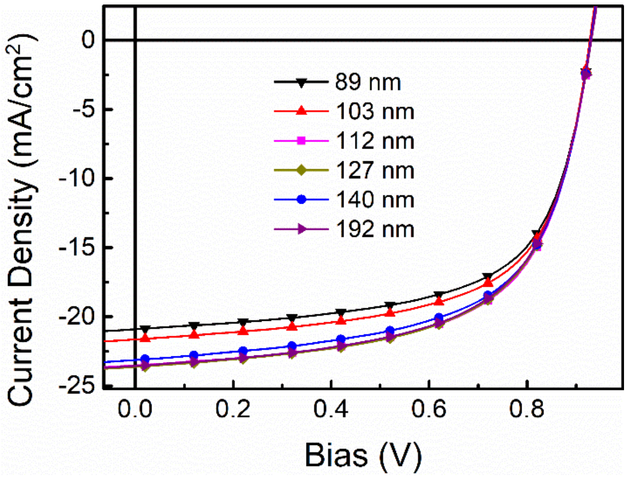

Figure S9. Photo-*J*–*V* curves based on PM6:**PYTT** blend device with different active layer thickness.

Table S2. Photovoltaic parameters of PM6:**PYTT** device with different active layer thickness

Thickness (nm)	V_{OC} (V)	J_{SC} (mA/cm^{-2})	FF (%)	PCE (%)
89	0.929 (0.929 ± 0.008)	20.90 (21.32 ± 0.40)	63.57 (65.32 ± 2.0)	12.35 (12.12 ± 0.23) [a]
103	0.928 (0.928 ± 0.012)	21.65 (22.10 ± 0.42)	63.18 (65.40 ± 2.7)	12.69 (12.37 ± 0.33) [a]
112	0.931 (0.940 ± 0.009)	23.52 (23.47 ± 0.38)	61.98 (63.40 ± 1.7)	13.58 (13.12 ± 0.46) [a]
127	0.931 (0.935 ± 0.004)	23.63 (23.10 ± 0.52)	61.54 (62.20 ± 3.0)	13.52 (13.34 ± 0.18) [a]
140	0.930 (0.936 ± 0.007)	23.12 (22.66 ± 0.46)	61.87 (61.34 ± 2.2)	13.31 (12.92 ± 0.39) [a]
192	0.931 (0.930 ± 0.01)	23.55 (23.26 ± 0.49)	61.31 (62.40 ± 1.9)	13.44 (13.32 ± 0.12) [a]

[a] The average parameters were calculated from six cells.

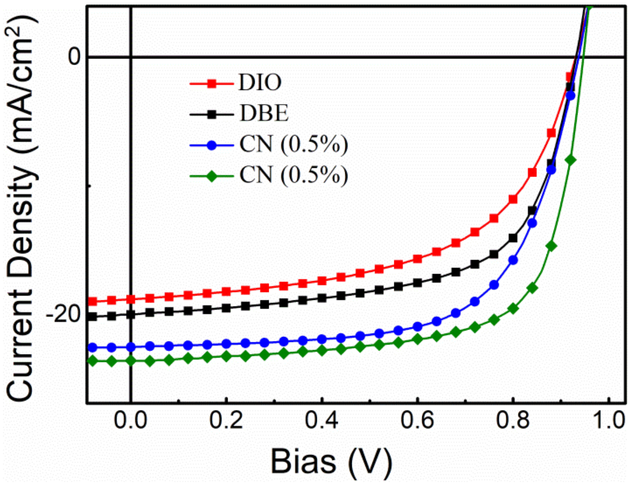

Figure S10. Photo-*J*–*V* curves based on PM6:**PYTT** blend device with various solvent additives.

Table S3. Photovoltaic parameters of PM6:**PYTT** device with different additives

**PM6:PYTT device**	V_{OC} (V)	J_{SC} (mA cm^{-2})	**FF** (%)	**PCE** (%)
CN (0.5%)[a]	0.942 (0.942 ± 0.06)	22.72 (22.89 ± 0.17)	66.26 (65.56 ± 0.77)	14.23 (13.33 ± 0.9) [c]
CN (0.5%)[b]	0.947 (0.942 ± 0.06)	23.42 (23.31 ± 0.45)	69.13 (68.26 ± 0.90)	15.32 (14.78 ± 0.54) [c]
DBE	0.935 (0.937 ± 0.008)	20.02 (20.77 ± 0.85)	63.26 (62.46 ± 0.97)	11.65 (11.03 ± 0.62) [c]
DIO	0.935 (0.932 ± 0.005)	18.83 (19.21 ± 0.42)	56.80 (56.25 ± 0.71)	9.84 (8.90 ± 0.94) [c]

[a] **PYTT** is extracted from the mixed fragments of dichloromethane (DCB) and CF; [b] **PYTT** is purified from the CF; [c] The average parameters were calculated from six cells.

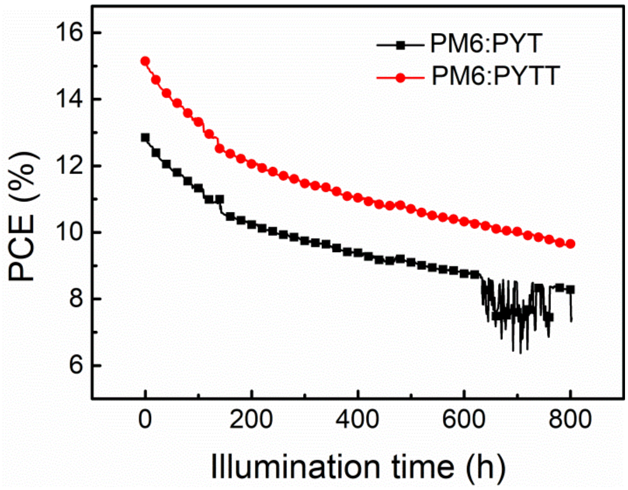

Figure S11. Photo-stabilities of PM6:**PYT** and PM6:**PYTT** blend devices under one sun light illumination.

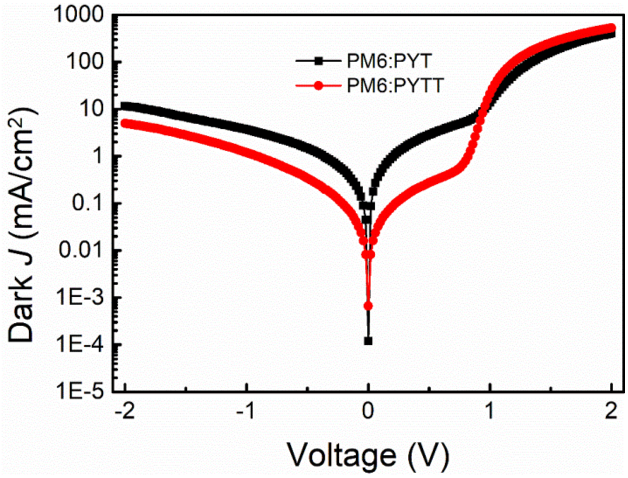

Figure S12. Dark *J*–*V* curves of PM6:PYT and PM6:**PYTT** blend devices.

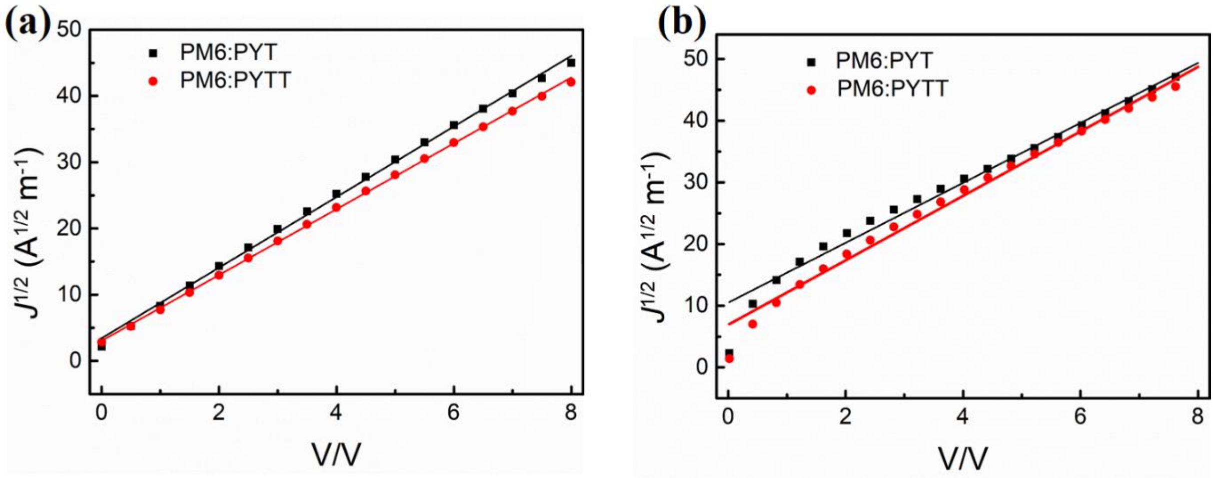

Figure S13. (a) Electron- and (b) hole-only devices based on PM6:PYT and PM6:**PYTT** blends.

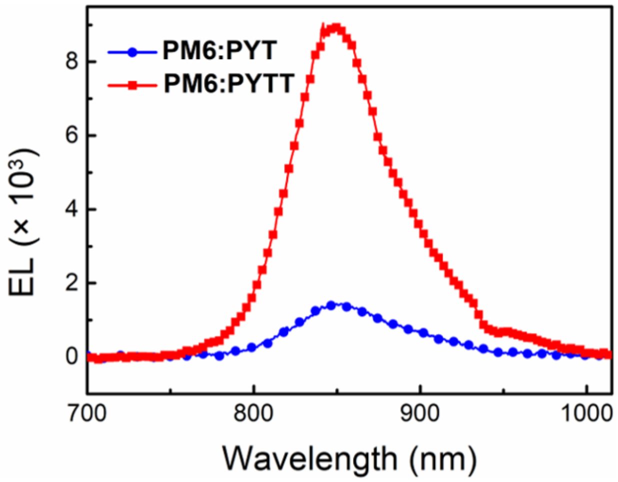

Figure S14. EL spectra of PM6:PYT and PM6:**PYTT** BHJ devices.

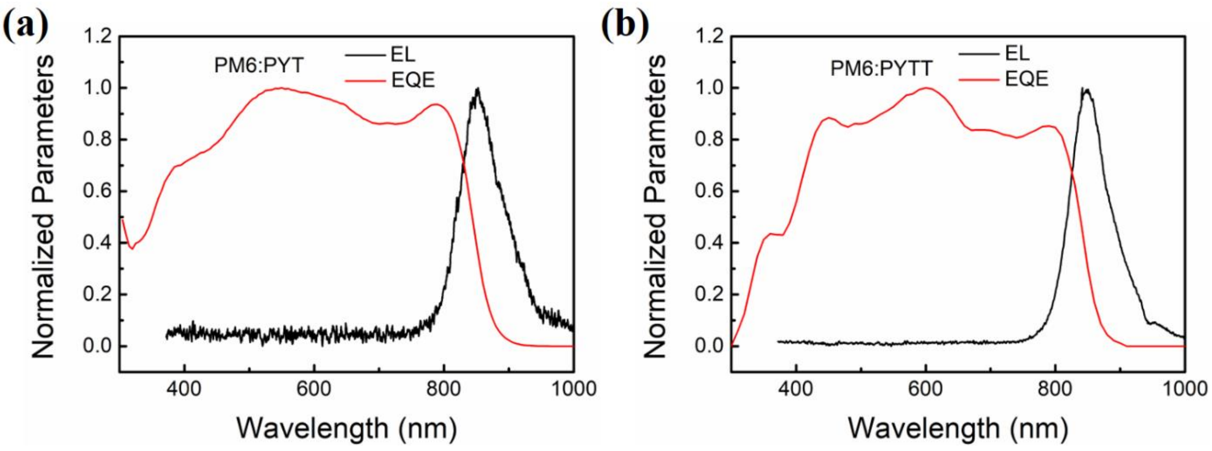

Figure S15. EL and EQE spectra of (a) PM6:PYT and (b) PM6:**PYTT** BHJ devices.

Table S4. Comparison of representative Y-system polymer acceptors based binary all-PSCs

Device	M_n (kDa)	M_w (kDa)	PDI	V_{OC} (V)	J_{SC} (mA cm^{-2})	FF (%)	PCE (%)	Reference
PBDB-T:PJ1-H	23.3	39	1.67	0.9	22.3	70	14.4	[S1]
PBDB-T:A701	21.1	39	1.85	0.92	18.27	64	10.7	[S2]
PM6:PYT$_M$	12.3	20.5	1.67	0.93	21.78	66.33	13.44	[S3]
PBDB-T:PTPBT-ET$_{0.3}$	18.33	32.6	1.78	0.9	21.33	65.3	12.52	[S4]
PM6:L14	19.04	38.68	2.03	0.96	20.6	72.1	14.3	[S5]
PBDB-T:PYE$_{20}$	13.8	172.5	12.5	0.91	20.97	71.63	13.6	[S6]
PM6:PYT-IT	20	40	2	0.93	22.3	72.3	15.05	[S7]
PTzBI-oF:PFA1	13.37	39.7	2.97	0.87	23.96	72.67	15.11	[S8]

PBDB-T:PF5-Y5	7.8	33.3	4.25	0.95	20.65	74	14.45	[S9]
PM6:PYF-T	11	24.4	2.22	0.88	23.27	66.83	13.77	[S10]
PBDB-T:PYTT-2	9.61	17.1	1.78	0.91	22	71.53	14.32	[S11]
PBDB-T:P(BDT2BOY 5-Cl)	14	25.2	1.8	0.92	18.72	63	11.12	[S12]
PBDB-T:PYN-BDTF	13.5	20.25	1.5	0.86	22.28	69	13.22	[S13]
PBDB-T:PZT-γ	7.8	16.38	2.1	0.90	24.7	71.3	15.8	[S14]
PBDB-T:PYT	9.1	15.7	1.73	0.89	22.64	70.02	14.06	[S15]
PM6:P2YFT	14.5	32.8	2.26	0.87	24.2	71.2	15	[S16]
JD40:PJ1	14.2	33.5	2.36	0.91	23.2	75	15.8	[S17]
PM6:PYF-T-o	11.1	19.87	1.79	0.90	23.3	72.4	15.2	[S18]
PBDB-T_{MW}:PJ1	11.4	22.8	2	0.90	22.2	75.3	15.4	[S19]
PBDB-T:PN-Se	11.7	32.76	2.8	0.91	24.82	71.8	16.16	[S20]
PM6:PY2F-T	14.5	32.77	2.26	0.86	24.27	72.62	15.22	[S21]
PBDB-T:PFY-3Se	34.0	52.36	1.54	0.87	23.6	0.737	15.1	[S22]
PM6:**PYTT**	28.2	43.12	1.54	0.95	23.67	69.80	**15.60**	**This work**

References

[S1] T. Jia, J. Zhang, W. Zhong, Y. Liang, K. Zhang, S. Dong, L. Ying, F. Liu, X. Wang, F. Huang, Y. Cao, *Nano Energy* **2020**, *72*, 104718.

[S2] A. Tang, J. Li, B. Zhang, J. Peng, E. Zhou, *ACS Macro Lett.* **2020**, *9*, 706.

[S3] W. Wang, Q. Wu, R. Sun, J. Guo, Y. Wu, M. Shi, W. Yang, H. Li, J. Min, *Joule* **2020**, *4*, 1070.

[S4] J. Du, K. Hu, L. Meng, I. Angunawela, J. Zhang, S. Qin, A. Liebman-Pelaez, C. Zhu, Z. Zhang, H. Ade, Y. Li, *Angew. Chem. Int. Ed*. **2020**, *59*, 15181.

[S5] H. Sun, H. Yu, Y. Shi, J. Yu, Z. Peng, X. Zhang, B. Liu, J. Wang, R. Singh, J. Lee, Y. Li, Z. Wei, Q. Liao, Z. Kan, L. Ye, H. Yan, F. Gao, X. Guo, *Adv. Mater*. **2020**, *32*, 2004183.

[S6] Y. Wu, Q. Wu, W. Wang, R. Sun, J. Min, *Sol. RRL* **2020**, *4*, 2000409.

[S7] Z. Luo, T. Liu, R. Ma, Y. Xiao, L. Zhan, G. Zhang, H. Sun, F. Ni, G. Chai, J. Wang, C. Zhong, Y. Zou, X. Guo, X. Lu, H. Chen, H. Yan, C. Yang, *Adv. Mater*. **2020**, *32*, 2005942.

[S8] F. Peng, K. An, W. Zhong, Z. Li, L. Ying, N. Li, Z. Huang, C. Zhu, B. Fan, F. Huang, Y. Cao, *ACS Energy Lett.* **2020**, *5*, 3702.
[S9] Q. Fan, Q. An, Y. Lin, Y. Xia, Q. Li, M. Zhang, W. Su, W. Peng, C. Zhang, F. Liu, L. Hou, W. Zhu, D. Yu, M. Xiao, E. Moons, F. Zhang, T. D. Anthopoulos, O. Inganäs, E. Wang, *Energy Environ. Sci.* **2020**, *13*, 5017.
[S10] H. Yu, Z. Qi, J. Yu, Y. Xiao, R. Sun, Z. Luo, A. M. H. Cheung, J. Zhang, H. Sun, W. Zhou, S. Chen, X. Guo, X. Lu, F. Gao, J. Min, H. Yan, *Adv. Energy Mater*. **2020**, *11*, 2003171.
[S11] T. Wang, R. Sun, W. Wang, H. Li, Y. Wu, J. Min, *Chem.Mater*. **2021**, *33*, 761.
[S12] J. W. Lee, C. Sun, B. S. Ma, H. J. Kim, C. Wang, J. M. Ryu, C. Lim, T. S. Kim, Y. H. Kim, S. K. Kwon, B. J. Kim, Efficient, *Adv. Energy Mater*. **2020**, *11*, 2003367.
[S13] N. Su, R. Ma, G. Li, T. Liu, L. W. Feng, C. Lin, J. Chen, J. Song, Y. Xiao, J. Qu, X. Lu, V. K. Sangwan, M. C. Hersam, H. Yan, A. Facchetti, T. J. Marks, *ACS Energy Lett.* **2021**, *6*, 728.
[S14] H. Fu, Y. Li, J. Yu, Z. Wu, Q. Fan, F. Lin, H. Y. Woo, F. Gao, Z. Zhu, A. K. Y. Jen, *J. Am. Chem. Soc*. **2021**, *143*, 2665.
[S15] Q. Wu, W. Wang, Y. Wu, Z. Chen, J. Guo, R. Sun, J. Guo, Y. Yang, J. Min, *Adv. Funct. Mater*. **2021**, *31*, 2010411.
[S16] R. Sun, W. Wang, H. Yu, Z. Chen, X. Xia, H. Shen, J. Guo, M. Shi, Y. Zheng, Y. Wu, W. Yang, T. Wang, Q. Wu, Y. Yang, X. Lu, J. Xia, C. J. Brabec, H. Yan, Y. Li, J. Min, *Joule* **2021**, *5*, 1548.
[S17] T. Jia, J. Zhang, K. Zhang, H. Tang, S. Dong, C.-H. Tan, X. Wang, F. Huang, *J. Mater.Chem. A* **2021**, *9*, 8975.
[S18] H. Yu, M. Pan, R. Sun, I. Agunawela, J. Zhang, Y. Li, Z. Qi, H. Han, X. Zou, W. Zhou, S. Chen, J. Y. L. Lai, S. Luo, Z. Luo, D. Zhao, X. Lu, H. Ade, F. Huang, J. Min, H. Yan, *Angew. Chem. Int. Ed.* **2021**, *60*, 10137.
[S19] L. Zhang, T. Jia, L. Pan, B. Wu, Z. Wang, K. Gao, F. Liu, C. Duan, F. Huang,Y. Cao, *Sci. China Chem.* **2021**, *64*, 408.
[S20] J. Du, K. Hu, J. Zhang, L. Meng, J. Yue, I. Angunawela, H. Yan, S. Qin, X. Kong, Z. Zhang, B. Guan, H. Ade, Y. Li, *Nat. Commun.* **2021**,*12*, 5264.
[S21] H. Yu, S. Luo, R. Sun, I. Angunawela, Z. Qi, Z. Peng, W. Zhou, H. Han, R. Wei, M. Pan, A. M. H. Cheung, D. Zhao, J. Zhang, H. Ade, J. Min, H. Yan, *Adv. Funct. Mater.* **2021**, *31*, 2100791.
[S22] Q. Fan, H. Fu, Q. Wu, Z. Wu, F. Lin, Z. Zhu, J. Min, H. Y. Wu, A. K. Y. Jen, *Angew. Chem. Int. Ed.* **2021**, *60*, 15935.
